# Dr. Andrew J. Jackson

**Published:** 1915-01

**Authors:** 


**Dr. Andrew J. Jackson,** Buffalo 1873, died at his home in Mattawan, N. J., November 17, 1914, aged 72.

